# Understanding stress: Insights from rodent models

**DOI:** 10.1016/j.crneur.2021.100013

**Published:** 2021-05-23

**Authors:** Fatin Atrooz, Karim A. Alkadhi, Samina Salim

**Affiliations:** Department of Pharmacological & Pharmaceutical Sciences, University of Houston, Houston, TX, USA

**Keywords:** Stress, Trauma, Mental health, Rodent behavior, Inflammation, Synaptic plasticity, ROS, reactive oxygen species, RNS, reactive nitrogen species, IL-6, interleukin 6, ERK_1/2_, extracellular signal-regulated kinase, CAMKIV, calcium/calmodulin-dependent protein kinase IV, CREB, cAMP response element binding protein, BDNF, brain-derived neurotrophic factor, NMDA, N-Methyl-D-aspartic acid, OF, open field, LD, light/dark, EPM, elevated plus maze, FST, forced swim test, CRP, C-reactive protein, TNFα, tumor necrosis factor alpha, HPA, hypothalamic–pituitary–adrenal axis

## Abstract

Through incorporating both physical and psychological forms of stressors, a variety of rodent models have provided important insights into the understanding of stress physiology. Rodent models also have provided significant information with regards to the mechanistic basis of the pathophysiology of stress-related disorders such as anxiety disorders, depressive illnesses, cognitive impairment and post-traumatic stress disorder. Additionally, rodent models of stress have served as valuable tools in the area of drug screening and drug development for treatment of stress-induced conditions. Although rodent models do not accurately reproduce the biochemical or physiological parameters of stress response and cannot fully mimic the natural progression of human disorders, yet, animal research has provided answers to many important scientific questions. In this review article, important studies utilizing a variety of stress models are described in terms of their design and apparatus, with specific focus on their capabilities to generate reliable behavioral and biochemical read-out. The review focusses on the utility of rodent models by discussing examples in the literature that offer important mechanistic insights into physiologically relevant questions. The review highlights the utility of rodent models of stress as important tools for advancing the mission of scientific research and inquiry.

## Highlights

• Stressful life events may lead to the onset of severe psychopathologies in humans.
• Rodents may model many features of stress exposure in human populations.
• Induction of stress via pharmacological and psychological manipulations alter rodent behavior.
• Mechanistic rodent studies reveal key molecular targets critical for new therapeutic targets.

## Graphical abstract

**Figure:**
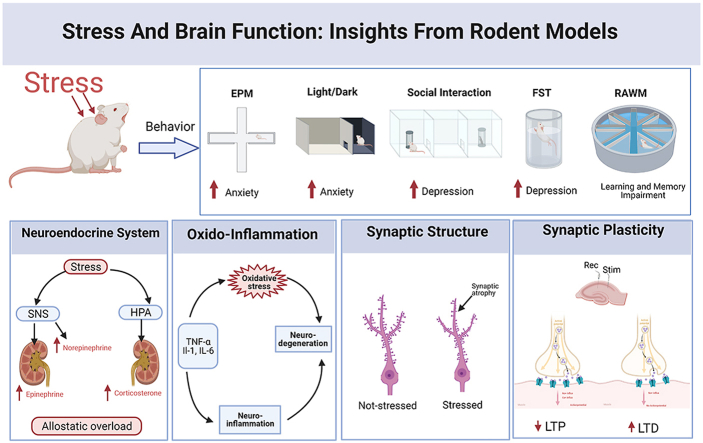

## 1 Introduction

Available animal models of stress partially mimic the stress-induced pathophysiological and behavioral changes as seen in humans (Jaggi et al., 2011). Based on the duration of stressor application, stress models can be categorized into acute or chronic models. In the acute model, stressor is applied once and for a short time, while chronic stress represents repeated application of stressful stimuli over an extended period (Bhatia et al., 2011). The term chronic, is generally considered relatively variable, therefore, in animal models often chronic stress is referred as prolonged stress. Animal models of stress can be further categorized as those that involve physical or psychological stressors. Physical stress involves potentially life-threatening bodily harm, such as the electric foot shock and forced swim stressors. While psychological stress does not produce physical pain per se but involves anticipation of physical pain, discomfort, or fear. Examples of psychological stressors include maternal separation, loud noises, immobilization, and predation (Bhatia et al., 2011). Different types of stressors often qualitatively present with differing behavioral and physiological patterns. For example, social defeat and electric foot shock are reported to produce differential responses on two major cardiovascular functions i.e. systolic blood pressure and mean arterial blood pressure. Social defeat produces increment in high blood pressure while foot shock reportedly causes decrement in blood pressure (Adams and Blizard, 1986). Examples of differential responses to various stressors such as physical versus psychological, suggest that biobehavioral studies of stress would be better served when focused on models that serve as stressors across mammalian species, including humans. A common animal trait is the struggle over resources associated with agonistic behaviors which often result in wounding, exhaustion, and death (Blanchard, McKittrick and Blanchard, 2001). Social stress, in addition to predatory stress, has provided great impetus for the evolution and understanding of stress mechanisms. Both social stress and predator stress are important, but social stress is a constant occurrence in the lives of higher social animals, while predator stress varies across species. Most laboratory studies examining the impact of social stress utilize rodents, rats or mice, and occasionally hamsters (Blanchard et al., 2001). Primates also serve as important subjects of laboratory studies investigating social stress effects, but their true social stress-related behaviors are more accurately observed under semi-natural conditions, or in the wild natural setting. In most mammalian species, hierarchies are clearly defined with more pronounced and dominant male hierarchies than females, thus male hierarchies tend to influence a broad spectrum of behaviors than females (Hannibal, Bliss-Moreau, Vandeleest, McCowan and Capitanio, 2017). It is for this reason, a number of different laboratory social stress models involve male rodents, although female rodents also are studied for maternal care and other behaviors (Blanchard et al., 2001).

The main components of the stress system are the HPA axis and the locus coeruleus-norepinephrine/autonomic systems. Activation of the stress system leads to behavioral and physiological changes responsible for adjustment of homeostasis (Chrousos and Gold, 1992). It has been widely reported that animal models of stress lead to a wide array of behavioral changes, especially emotionality-linked behaviors like anxiety, defensiveness, substance self-administration, and reduction in social interactions and sexual behaviors (Blanchard et al., 2001). Exposure to stressful events also can alter important cognitive functions like learning and memory processes (Kim and Diamond, 2002, Kim and Diamond, 2002). Stress paradigms reportedly produce several critical modifications in the brain, causing structural and functional impairments by altering neuronal structure, cell survival and neurotransmission. Generally, stress paradigms are believed to modify various dopaminergic, GABAergic, and excitatory amino acid transmission. In neuropeptide systems, corticotropin-releasing factor (CRF) and hormone vasopressin (AVP) pathways of the HPA axis are activated by stress, while extra-hypothalamic AVP and CRF circuits are inhibited and stimulated, respectively (R Adamec, Kent, Anisman, Shallow and Merali, 1998; Anisman, Prakash, Merali and Poulter, 2007). Chronic social stress is reported to alter key morphological changes within the hippocampal neurons, which may significantly alter learning and memory processes and functions (Bruce S McEwen, 1999, McEwen, 1999). Hippocampal mediated learning and memory also are reduced in stressed animals. Stressful stimuli in animals also reduce neurogenesis within the dentate gyrus (B. S. McEwen, 2001). Several studies have reported involvement of chronic stress in hippocampal neuron formation, which is believed to cause alterations in dendritic morphology, cell survival, and neurogenesis. Significant shrinkage in the apical dendritic arbors of CA3 pyramidal neurons has been reported suggesting that dendritic remodeling is a well-supported response to chronic HPA axis activation (A. M. Magariños, Deslandes and McEwen, 1999; Magarinos and McEwen, 1995). Similar dendritic remodeling also has been reported in the hippocampus of subordinate tree shrews (A. M. a. Magariños, McEwen, Flügge and Fuchs, 1996). Although neuronal survival is not directly modified in psychosocial stress, a study in tree shrews has suggested that neurogenesis is inhibited within the dentate gyrus of subordinate tree shrews when compared with control tree shews (Elizabeth Gould, Bruce S McEwen, Patima Tanapat, Liisa AM Galea and Eberhard Fuchs, 1997). Evidence of neurodegeneration were observed in the hippocampus of vervet monkeys who died from stress related causes in primate center in Kenya, the monkeys exhibited stressful phenotype, including gastric ulcers, enlarged adrenals, and signs of social conflict, evident from bite marks (Uno, Tarara, Else, Suleman and Sapolsky, 1989). The effects of stress on both pre- and postsynaptic components of noradrenergic neurotransmission have been extensively studied (Stone and Quartermain, 1999; Valentino, Foote and Page, 1993). A variety of stressful events, reportedly, cause a significant increase in noradrenaline release in several brain regions, particularly within the hypothalamus, amygdala and the locus coeruleus (LC) areas of the brain (Anda H Van Stegeren, 2008). These findings suggest that an increased noradrenaline release is most likely linked with the provocation of negative emotions of anxiety and/or fear. (Tanaka, Yoshida, Emoto and Ishii, 2000). Stressful stimuli is reported to selectively causing increased expression of tyrosine hydroxylase (TH), the rate-limiting enzyme in catecholamine synthesis, as indicated by the increase in TH mRNA in noradrenergic brain areas (Brady, Blanchard and Blanchard, 1994). The increased mRNA levels in the LC region was reported to be accompanied with a corresponding increase in immunoreactive TH protein (Brady et al., 1994; Watanabe et al., 1995). Several different stress paradigms have shown activation of the LC noradrenergic system (Stanford, 1995). It is postulated that the changes in TH are indicative of an upregulation of the capacity of synthesis due to increased neuronal activity and neurotransmitter release. Stress-induced changes in adrenergic receptors also have been reported (Flügge, 1996; Flügge, Ahrens and Fuchs, 1997; Flügge, Jöhren and Fuchs, 1992), suggesting that intricate changes in the regional populations of adrenergic receptor subtypes may differentially regulate the function of various noradrenergic circuits as a stress response mechanism. Furthermore, acute or chronic stress may also differentially regulate receptor turnover, and synthesis (Blanchard et al., 2001). Several key behaviors such as anxiety-like behavior, depression-like behavior and social behavior are affected by stress paradigms. *Anxiety-Like Behavior*: Anxiety disorders are characterized by excessive fear and avoidance to specific objects or situations when little or no danger present (Shin and Liberzon, 2010). Anxiety is frequently linked with stress exposure; this link is highlighted by the reported overlap between the neurochemical mechanisms activated by exposure to stressful events and those involved in the regulation of anxiety (Patchev and Patchev, 2006). The development of animal models of anxiety and models of stress have helped to understand the pathophysiology of stress disorders and to identify potential therapeutic effects of novel drugs. Rodent models of anxiety involve environmental manipulations that can alter anxiety levels. Such manipulations include exposing rodents to a form of stress followed by assessment of anxiety-like behaviors. Evaluation of anxiety levels in rodent models of stress generally focus on ethologically relevant behavioral paradigms, which are described as anxiety-like behaviors. The basic premise of these tests is creating a typical approach-avoidance conflict by exposing the rodents to a new environment, which initiates simultaneous bouts of fear and curiosity, creating behavioral responses that are correlated with anxiety-like behavior which resemble anxiety behaviors in humans, though in rodents they might actually represent something else (Lezak, Missig and Carlezon Jr, 2017). (Campos, Fogaça, Aguiar and Guimaraes, 2013). Example of approach/avoidance models used for examination of anxiety-like behavior in rodents are: light/dark box, elevated plus maze, open field, and novelty suppressed feeding tests (Lezak et al., 2017; Patki et al., 2015, Patki et al., 2015; Gaurav Patki et al., 2014, Patki et al., 2014, Patki et al., 2014). *Depression-Like Behavior*: Depression is a common mental disorder characterized by persistent low mood or negative affect with a concomitant expression of reduced positive affect also described as anhedonia. Stress is recognized as a common risk factor for depression (Rincón-Cortés and Grace, 2020). A variety of animal models have been designed over the years, with the goal to help elucidate the pathophysiology underlyng depression and to test efficacy of novel antidepressant treatment strategies. Despite considerable efforts, the currently available animal models only partially mimic this complex disorder in humans. Compared with acute stress paradigms, chronic paradigms, like the chronic restraint stress, chronic social defeat stress and chronic unpredictable mild stress, showed more reliability and effectiveness in modeling depression-like behavior in rodents (Antoniuk, Bijata, Ponimaskin and Wlodarczyk, 2019; Qiao et al., 2016). Additionally, early life stress paradigms such as maternal separation and single prolonged stress effectively induced depression-like behavior in susceptible but not resilient rodents, suggesting that other aspects such as genetic factors might modulate stress vulnerability (Czéh, Fuchs, Wiborg and Simon, 2016; Liu, Atrooz, Salvi and Salim, 2017; Murthy and Gould, 2018). Assessment of depression-like behavior in rodents involves anhedonia-like behaviors, reward-seeking behaviors, and active- or passive-coping behavioral patterns (Czéh et al., 2016). Anhedonia is a core symptom of depression in humans, it is defined as the inability to experience pleasure from rewarding or enjoyable activities. The primary readouts for anhedonia-like behavior is sucrose preference or consumption. The place preference paradigm has been used extensively to study the rewarding properties of drug abuse, it has also been used to assess the tendency to natural rewards such as food or sweet solutions (Papp, Willner and Muscat, 1991). Active- or passive-coping behavior involves placing the rodents in inescapable challenging environment and record their behavior. Forced swim test (FST) and tail suspension test are examples of passive-coping behaviors that are used for assessment of depression-like behavior in rodents (Cryan, Mombereau and Vassout, 2005; Porsolt, Le Pichon and Jalfre, 1977). A consistent finding while using chronic stress exposure in rodents is described as the occurrence of a disruption in the brain reward system. The network of structures comprising the brain reward circuit includes the ventral tegmental area (VTA), ventromedial prefrontal cortex, orbitofrontal cortex, amygdala, hippocampus, nucleus accumbens, anterior cingulate cortex, ventral pallidum and associated structures (Der-Avakian and Markou, 2012; Rizvi, Pizzagalli, Sproule and Kennedy, 2016). Among all, a well-researched component of the brain reward circuitry is the mesolimbic dopaminergic system, which projects from the VTA to the nucleus accumbens. The dopaminergic pathway is involved in motivational aspects and processing of reward-related stimuli. Activation of the mesolimbic dopaminergic system by rewarding stimuli such as food or administration of drugs of abuse, induces dopamine release within the nucleus accumbens (Schultz, 2016; D. A. Slattery and Cryan, 2017). *Social Behavior***:** The neurobiology of stress response overlaps with the neurobiology of social behavior. While negative social interactions can be potent stressors, social support can buffer the response to an external stressor, though, social behavior often changes in response to stressful life experience (Beery and Kaufer, 2015). Different stress paradigms have been reported to induce social avoidance and social fear in rodents; those paradigms involve non-social (physical) or social stressors or a combination of both. However, social stress paradigms or a combination of social and non-social stressors more accurately mimic the human clinical situation and thus are considered as one of the most translationally relevant approaches. To assess social behavior in rodents, many behavioral tests have been designed on the premise that rodents prefer to spend time with another animal rather than remaining alone or to engage in exploration of social rather than non-social stimuli (File and Hyde, 1978; Lukas et al., 2011; Toth and Neumann, 2013). The neuropeptides oxytocin (OXT) and arginine vasopressin (AVP) are known to play a role in social bonding. In rodents’ brain, OXT is suggested to stimulate social behavior by inhibiting social anxiety and defense which is demonstrated by facilitation of approach behaviors (K. A. Young, Liu and Wang, 2008). Moreover, OXT is believed to play an important role in reducing stress by dampening HPA-activity, producing anxiolytic-like effects (Bartz and Hollander, 2006). AVP is predominantly involves in male social behavior (e.g. aggression, scent marking, courtship), stress adaptation and anxiety, and maternal nurturing behavior (Burbach, Young and Russell, 2006; Lim and Young, 2006). During social interactions, the enhanced release of OXT and AVP might establish the hedonic value of social contact through activation of the reward system (Love, 2014; L. J. Young, 2002). Some studies suggested that acute stress increases central OXT secretion. For instance, novelty and restraint stress increased OXT levels in plasma and cerebrospinal fluid (Ivanyi, Wiegant and De Wied, 1991; Wotjak et al., 1996).

The chronic subordinate colony (CSC) housing and social isolation paradigms have been also used to induce social avoidance in rodents. The CSC paradigm increases anxiety-like behavior as reflected from social avoidance behavior in a consistent and reliable manner (David A Slattery et al., 2012). The social isolation paradigm also used as a model of social stress. Studies showed that both male and female rats, especially when socially isolated between postnatal days 22 and 35, a period considered critical for development of sociability, showed impaired social interaction and social avoidance behaviors. Social isolation during critical period of postnatal brain development induced anxiety-like behavior and social avoidance associated with reduced expression of synaptic-associated proteins in the prefrontal cortex (Hermes, Li, Duman and Duman, 2011). Non-social stress paradigms such as inescapable foot shock has been shown to induce social avoidance in the social interaction test and social approach-avoidance test (Louvart, Maccari, Ducrocq, Thomas and Darnaudéry, 2005; Mikics et al., 2008; Short and Maier, 1993). Additionally, both acute and chronic restraint stress paradigm reportedly induce avoidance behaviors in both rats and mice (Gameiro et al., 2006; Manchanda, Jaggi and Singh, 2011). A growing body of literature demonstrating that stress is a potent modulator of learning and memory processes. Therefore, assessment of learning and memory can be used for examination of both transient and persistent consequences of induction of stress. (Patchev and Patchev, 2006). Several rodent models of stress have suggested that chronic stress was associated with learning and memory deficits (A. Aleisa, Alzoubi, Gerges and Alkadhi, 2006; Alzoubi et al., 2009, Alzoubi et al., 2009; Antonova and Mueller, 2008; Gaurav Patki, Lumeng Li et al., 2014; Gaurav Patki, Naimesh Solanki and Samina Salim, 2014; Solanki, Alkadhi, Atrooz, Patki and Salim, 2015). For assessment of cognitive function in rodents, both spatial- and non-spatial- memory tasks were employed. Spatial memory is commonly defined as the brain function responsible for recognition, storage, and recovery of spatial information with regards to the arrangement of objects or paths. Different type of mazes has been employed for evaluation of spatial memory in rodents including the radial arm maze, radial arm water maze, Morris water maze, and Y-maze. The fundamental assumption for the use of these mazes is based on the behavioral principle that animals should learn and remember the location that provides them with safety, food, or other reward by means of visual–spatial signals (Paul, Magda and Abel, 2009). Non-spatial memory tasks included object recognition memory and temporal order recognition memory. The novel object recognition (NOR) task is one of the most commonly used behavioral paradigms used for assessment of memory. The is task based upon the innate preference of the rodents to explore novel object versus the familiar one. Therefore, the animal which remembers the familiar object will end up spending overall more time exploring via sniffing, circling, touching and palying with the novel object (Broadbent, Gaskin, Squire and Clark, 2010; Ennaceur, Neave and Aggleton, 1997; Leger et al., 2013).

Several publications, based on animal models, proposed dichotomous effects of stress on learning and memory functions. Short and controllable stress reportedly facilitates memory acquisition, while severe chronic persistent stress interferes with memory consolidation and memory retrieval processes (de Kloet, Oitzl and Joëls, 1999; Joëls, Pu, Wiegert, Oitzl and Krugers, 2006). Enhanced memories resulting from stressful learning situations can be related to the effect of stress on memory acquisition and subsequent consolidation of information. Mmeory acquisition facilitation can be achieved by altering a variety of psychobiological functions including attention, motivation, sensory processing, integration of sensory inputs, and motor functions (Roozendaal, 2002, 2003). In fact, stress is considered as a critical component of emotional modulation of memory, therefore, typically emotionally arousing experiences are better preserved and remembered than neutral ones (Cahill and McGaugh, 1998; James L McGaugh, 2004, McGaugh, 2004; Sandi, 1998).

On the other hand, severe or chronic stress is suggested to induce alterations of cognitive functions including working memory, attention, and reward processing (Arnsten, 2009, 2015). However, accumulated data show that stress does not elicit identical effect in males and females. While chronic stressors in rodents impair male cognitive functions in spatial memory tasks, females show cognitive resilience in chronic stress paradigms (Luine, Gomez, Beck and Bowman, 2017). Furthermore, females receiving restraint stress showed enhanced spatial memory, as assessed by the object placement test when compared to control females (Beck and Luine, 2002; Bisagno et al., 2004). An interesting postulation is that estrogens might provide cognitive resilience to stress in females (Luine et al., 2017).

Substantial body of research using rodent models demonstrates that exposure to stress causes structural remodeling of the principal projection neurons within the prefrontal cortex (PFC). Notably, alterations in the PFC neuronal morphology are reported to be associated with deficits in rodent executive functions including working memory, attentional set-shifting and cognitive flexibility functions (Holmes et al., 2009). Brain activity studies showed that the PFC in rodents is highly activated by a variety of stressors (Campeau, Dolan, Akil and Watson, 2002; Cullinan, Herman, Battaglia, Akil and Watson, 1995; Morrow, Elsworth, Lee and Roth, 2000). Activation of PFC during exposure to aversive stimuli is most likely indicative of the implication of PFC as a modulator of the neuroendocrine stress-response system (Herman, Ostrander, Mueller and Figueiredo, 2005). Different animal models of stress have proposed a pivotal role of the PFC in maladaptive responses to stress. For instance, exposure to unpredictable stress caused dendritic atrophy in the PFC associated with impaired spatial working memory as indicated by Morris water maze test (Cerqueira, Mailliet, Almeida, Jay and Sousa, 2007). In a different study, brief exposure to restraint stress produced significant effect on the cognitive functions of the PFC as indicated by spatial delayed alternation task (Shansky, Rubinow, Brennan and Arnsten, 2006). Below, we discuss some of the widely used rodent models of stress.

### 1.1 Rodent models of stress

***Psychosocial Stress:*** Social contact and social interactions are considered a source of stress, which is evolutionarily preserved and also ubiquitous among mammalian species. The nature, extent and intensity of stressful experiences however vary depending upon the species, sex, and age. A unifying theme among all social stress models is the fact that social stress strongly impacts behavior, with some exhibiting aggression, victorious or dominance, while others exhibiting defensiveness, defeat, or subordinate behaviors. In addition to behavioral effects, the effects of social stress also have been reported to impact brain neurotransmitter systems especially the serotonin and noradrenergic systems. Neuroanatomical and a variety of molecular changes have been reported with severe social stress, as it induces long-term changes in hypothalamic-pituitary-adrenal (HPA) axis functioning. Thus, the evidence clearly suggests that social stress models serve as appropriate stressors for research, the interpretation of the findings however must be cautiously drawn as some behaviors in subordinate animals might be affected by the presence of a dominant rather than representing behavioral alteration (Blanchard et al., 2001).

***Intruder chronic psychosocial stress:***This model involves stressing two groups of rats kept in two separate cages (4–6 rats/cage). Rats in each group are housed with same cage mates for at least 1 week to enable social hierarchy establishment within the group. (Gerges and Alkadhi, 2004). Then, psychosocial stress is induced by a daily randomized switching pattern by switching 2 rats from one cage to another, for a total period of 6 weeks. This switching disrupts the social hierarchy, forcing rats to continuously readjust to new stressful conditions. The rat intruder model of psychosocial stress mimics psychosocial stress in humans, as encountered at home or at the work place, and has been shown to increase plasma corticosterone levels (Gerges and Alkadhi, 2004) and also significantly elevate rat blood pressure parameters (Karim A Alkadhi, Alzoubi, Aleisa, Tanner and Nimer, 2005).

***Social Defeat Paradigm:*** This model is known as the resident-intruder model originally which was originally developed by Miczek and colleagues (Miczek, 1979). The social defeat model mimics some aspects of societal stress as experienced by humans. The social defeat paradigm is postulated to model some aspects of human aggression, bullying, chronic subordination and experiences of humiliation which are often believed to contribute to the development of post-traumatic stress disorder (PTSD) (Björkqvist, 2001; Rohde, 2001). As previously published (G. Patki, N. Solanki, & S. Salim, 2014), this paradigm consists of 7 encounters between an aggressive male Long Evans (LE) rat and an intruder Sprague Dawley rat. After acclimatization in home cages for at least one week, the session is initiated by placing the intruder rat in the resident LE rat home cage for 30 ​min. The resident rat engages in repeated attacks on the intruder. A typical social defeat is indicated by the intruder surrendering or by acquiring a supine position over beneath the intruder LE rat for approximately 3 ​s. Once the intruder rat is defeated, a perforated Plexiglas partition is placed in the middle of the cage to avoid direct physical contact between the LE rat and the defeated Sprague Dawley rat. While the perforated partition prevents the direct physical contact between the rats, it allows intense visual, auditory, and olfactory interactions for the remainder of the 30-min session. If the resident LE rat struggles to defeat the intruder for the first 10 ​min of the session, rats are separated with the help of a partition for the remainder 20-min of the session. Three sessions are carried out for seven successive days, each test rat should encounter different resident LE rat every day. Control rats are placed behind a Plexiglas partition in a fresh cage for 30 ​min daily. Rats are returned to their home cage after each social defeat session. Rats can be tested for behavior 24 ​h following the last session. While this paradigm is mainly used in male animals, some researchers successfully used it for acute or chronic psychosocial stress in female rodents by employing lactating females as residents as they display high level of maternal aggression towards intruders (Neumann, Toschi, Ohl, Torner and Krömer, 2001). Socially defeated rats reportedly exhibit significant physiological and behavioral changes including; increased activity of HPA axis, sensitivity to other stressors, anxiety-like behavior, and depression-like behavior (Hollis and Kabbaj, 2014; Gaurav Patki, Lumeng Li et al., 2014). (Macedo et al., 2018; Gaurav Patki, Lumeng Li et al., 2014; Patki, Solanki, Atrooz, Allam and Salim, 2013). Most studies on social defeat stress suggested activation of HPA-axis stress system indicated by elevation of plasma corticosterone (CORT) levels following social defeat (Becker et al., 2008; Choleris, Clipperton-Allen, Phan and Kavaliers, 2009; Solanki, Salvi, Patki and Salim, 2017). Social defeat stress also induced alteration in monoamine levels in stress-responsive limbic brain regions; socially defeated adolescent rats exhibited anxiety-like behaviors at adulthood that were associated with decreased medial prefrontal cortex dopamine, elevated levels of norepinephrine and serotonin in the ventral dentate gyrus region of the brain, combined with decreased levels of norepinephrine in the dorsal raphe region (Watt, Burke, Renner and Forster, 2009). Repeated social defeat paradigms using the resident/intruder paradigm, induces depressive-like behaviors such as social avoidance when presented to an unfamiliar conspecific animal and reduced sucrose preference. Moreover, chronic social defeat induced behavior despair as indicated by increased immobility in FST and tail suspension tests (Kudryavtseva, Madorskaya and Bakshtanovskaya, 1991; Rygula et al., 2005). Importantly, other studies reported presence of two groups following chronic social stress; susceptible vs resilient: susceptible rodents exhibited social avoidance and enduring anhedonia-like phenotype, behavior alteration were accompanied by persistently decreased brain reward function demonstrated by alterations in the firing rates of VTA dopaminergic neurons, whereas resilient rodents showed no long-term brain reward deficits (Der-Avakian, Mazei-Robison, Kesby, Nestler and Markou, 2014; Krishnan et al., 2007). Several studies have demonstrated that expression of OXT and AVP were enhanced after a single social defeat (Ebner, Wotjak, Landgraf and Engelmann, 2000; Engelmann, Ebner, Landgraf, Holsboer and Wotjak, 1999; Wotjak et al., 1996). In fact, some evidence suggested that enhanced OXT and AVP release might moderate the social behavioral response to social defeat in mammals and protect defeated individuals against stress-induced pathological sequalae (Lukas et al., 2011; Nishioka, Anselmo-Franci, Li, Callahan and Morris, 1998). However, induction of chronic social defeat in adult females voles reduced levels of OXT and OXT receptors which also was associated with reduced occureence of social preference behaviors in these animals (L. Wang et al., 2018). In parallel, other studies reported that chronic or severe stress exposure to social defeat resulted in occureence of significantly prolonged social avoidance in both mice and rats (O. Berton et al., 2006; Hammels et al., 2015; Hollis and Kabbaj, 2014; Hollis, Wang, Dietz, Gunjan and Kabbaj, 2010). Interestingly, Krishnan and his colleague (Krishnan et al., 2007) reported that according to social motivation after exposure to social defeat stress, two groups of mice were identified, susceptible and resilient. Susceptibility to stress is considered to be closely related to stress-induced increase in levels of the brain derived neurotrophic factor (BDNF) within the nucleus accumbens (NAc) region of the brain. BDNF is believed to be a key regulator of dopamine release in the NAc-ventral tegmental area (VTA) of the brain. The findings suggested that susceptibility to social defeat might be enabled via enhanced firing of VTA dopamine neurons, while resilience to stress may be characterized by a lack of activity-dependent BDNF release (Krishnan et al., 2007).

***Restraint Stress:*** Restraint stress is produced by placing the animal in a small Plexiglas cylinder with perforation walls, to allow normal breathing. The perforated Plexiglas cylinder is adjusted according to animal body weight, so that the animal is fully immobilized. Animals also can be restrained by placing them individually with compression in double layered plastic Ziploc bag while edges wrapped firmly with duct tape. Restraint can be induced once for 2 ​h to produce acute stress, or for 7–14 days to produce chronic stress. The major advantage of the restraint stress is that it produces an inescapable physical and psychological stress to which adaptation is rarely developed (Bhatia et al., 2011; Marin, Cruz and Planeta, 2007).

***Chronic Unpredictable Stress (CUS):*** A major disadvantage of most animal models of stress is development of adaptation and/or resistance on long-term stress exposure. CUS models are particularly useful in this regard due to the application of a variety of unpredictable stressors used to create unpredictable stresses CUS. Another important advantage of CUS model is the development of effective and long-lasting stress responses even after a long gap of the application of the stressors (Bhatia et al., 2011). CUS is a routinely used model in animal studies of depression (Larsen, Mikkelsen, Hay-Schmidt and Sandi, 2010). In this model, the animals are exposed to different types of mild to moderate physical and psychological stressors on a variable basis to prevent adaptation or resistance to a single kind of stressor. Animals are exposed to various mild to moderate stressors one by one on daily basis for 17 or 21 days. For example, in one protocol, animals are exposed to restraint stressor for 15 ​min followed by overnight sleep deprivation, on day 2, they are subjected to rotation of the cage at a predetermined high speed for 50 ​min followed by swim stress in water at room temperature for 4 ​min (Kanarik et al., 2008). Another protocol of CUS includes wetting the bedding of the animals throughout the day to restrict their movement followed by electric foot shock (ten shocks of 0.4–1.8 ​mA, each for 1 ​s duration), then application of stroboscopic light (10 ​Hz) for 13 ​h (Anisman et al., 2007). Other stressors that are used in the CUS model include; exposure to predator odor, strong illumination during dark phase for 12 ​h, tail pinch with a clothes-pin placed 1 ​cm distal from the base of the tail for 5 ​min, movement restriction in a small cage for 2 ​h, cold water (4C), ether anesthesia until loss of consciousness, and subcutaneous 0.9% saline injection, among others (Kanarik et al., 2008; Ladd, Thrivikraman, Huot and Plotsky, 2005). Chronic restraint stress has been reported to induce depression-like behaviors in rodents (Sevgi, Ozek and Eroglu, 2006; X. Wang et al., 2016; P. Xu et al., 2017). Depression-like behavior was associated with decreased expression of annexin II light chain (p11), a multifunctional protein that binds to 5-HT receptors, in layer II/III neurons of the prelimbic cortex. Interestingly, both; behavior deficits and P11 loss were reversed by treatment with selective serotonin reuptake inhibitors (SSRIs) and the tricyclic class of antidepressant (TCA) agents. The authors have suggested that p11 protein as a cell-type specific molecule that regulates stress-induced depression (Seo et al., 2017). Models of chronic stress in rodents demonstrated that chronic stress exposure causes disruption of the brain reward system as indexed by anhedonia and despair-like phenotypes (Chang and Grace, 2014; Moreau, Jenck, Martin, Mortas and Haefely, 1992). CUS has been shown to induce anhedonia and a decreased response to various types of rewards in rodents, suggesting alterations in dopaminergic reward system (Scheggi, De Montis and Gambarana, 2018; Paul Willner, Muscat and Papp, 1992). For example, mice and rats exposed to CUS exhibited an anhedonia-like state associated with decreased reward responsiveness indicated by reduced sucrose preference and consumption, attenuation of conditioned place preference, decreased sexual activity, and increased thresholds for intracranial self-stimulation in the VTA. Additionally, CUS induced depression-related phenotypes in rodent measures of behavioral despair, which can also be interpreted as passive coping, indicated by increased immobility in FST and tail suspension test. Notably, antidepressant treatment reverted behavioral changes induced by CUS (Grønli et al., 2005; Monleon et al., 1995; Moreau et al., 1992; Paolo, Brain and Willner, 1994; P Willner, 1995; Paul Willner, Towell, Sampson, Sophokleous and Muscat, 1987). In a mouse model of early life unpredictable stress (UPS), mice exposed to UPS exhibited robust increase in anxiety-like behavior during juvenile and adult developmental stages. Interestingly, using fMRI imaging, UPS adult mice showed increased amygdala–prefrontal cortex and amygdala–hippocampus connectivity as compared to control mice (Johnson et al., 2018). In male rats and mice, exposure to chronic mild stress (CMS), repeated restraint stress or CUS, impaired cognitive performance as indicated by spatial memory function tests (Li et al., 2008) (Cheryl D Conrad, 2010; Nishimura, Endo and Kimura, 1999; Song, Che, Min-Wei, Murakami and Matsumoto, 2006), for review, see (Conrad, 2010). Though, other studies suggested that learning and memory is enhanced in females following chronic stress. Female rats exposed to repeated restraint stress showed improvement in the performance in two kinds of maze models including radial arm water maze and Morris water maze (Bowman, Zrull and Luine, 2001; Kitraki, Kremmyda, Youlatos, Alexis and Kittas, 2004).

***Chronic Subordinate Colony Housing Paradigm (CSC):*** The CSC paradigm involves long-term induction of psychological and social stress. CSC is believed to mimic the stressors faced by humans (Langgartner, Füchsl, Uschold-Schmidt, Slattery and Reber, 2015). In this paradigm, CSC mice are housed in prolonged subordination of a dominant resident mouse for 19 consecutive days (S. Reber et al., 2007). Shortly after placing the CSC mouse into the large male resident's cage, resident mouse assumes a dominant position by exhibiting offensive behaviors toward the CSC mouse. Aggressive behavior is displayed in the form of chasing, mounting, or attacking CSC mice. In contrast, CSC mice are considered as “subordinates” based on demonstration of their defensive behaviors, such as flight response and submissive behavior (S. O. Reber and Neumann, 2008).

***Trauma Witness (TW):*** Witnessing a traumatic event increases vulnerability to psychiatric symptoms including PTSD (Blanchard et al., 2001). Therefore, different animal models have been developed to examine the consequences of witnessing traumatic events (Patki, Salvi, Liu and Salim, 2015; Warren et al., 2013). In a rat model of trauma witness, social defeat sessions are observed by the trauma witness (TW) rats present outside the resident (LE) cage. After placing the intruder rat in the resident LE rat cage, TW rats are placed outside the resident's cage to observe social defeat. It is reported that TW rats initiate a freezing response while they witness the social defeat (Gaurav Patki, Naimesh Solanki et al., 2014). During each session, TW rats witness three bouts of social defeat with 5-min separation between the bouts, in order to reinforce the visual stress in the TW rats. For control rats, another intruder rat is placed behind a wire partition in a novel cage as social defeat control, the TW control rats are placed outside the cage for 30 ​min daily. Trauma witness is reported to induce anxiety- and depression-like behavior in rats, it also causes learning and memory deficits. Interestingly, social housing significantly mitigated the behavioral deficits in TW rats as compared to solitary housed rats following trauma witness (G. Patki et al., 2014).

***Early Life Maternal Trauma Witness:*** Witnessing maternal abuse reportedly induces helplessness and development of behavioral deficits and depression in children (Seng et al., 2013; Stirling and Amaya-Jackson, 2008). Maternal trauma witness was modeled in rats (Liu, Patki, Salvi, Kelly and Salim, 2018). In this paradigm, pups are allowed to witness their mother get exposed to social defeat. The apparatus is designed to include a central enclosure for social defeat, with six separate small witness enclosures. Pups of natural litters of the dam are exposed to daily witnessing of repeated social defeat (attacks) of their mother by an unfamiliar aggressive LE male rat for 45 ​min (3 intervals, 10 ​min of social defeat followed by 5 ​min of rest), for 7 consecutive days, after conclusion of social defeat witness protocol, pups are left undisturbed for one month in their home cages before conducting behavior assessment. Control exposures is done by subjecting a female rats to a novel cage without the presence of male LE rat in the presence of pups around the cage, the pups are placed in transparent separate enclosures for 30 ​min daily exposure for 7 days. Witnessing repeated maternal traumatic exposure caused depression-like behavior in both male and female rats at postnatal day (PND) 60, Interestingly, socially defeated female rats, the mother of pups who witnessed social defeat, developed both anxiety and depression-like behavior (Liu et al., 2018).

***Single Prolonged Stress (SPS):*** This paradigm represents acute stress. It involves combined stress paradigms applied consecutively in one day (Liberzon and Young, 1997; Perrine et al., 2016). First, the animals are exposed to immobilization for 2 ​h by placing them individually with compression in double layered plastic Ziploc bag while edges wrapped firmly with duct tape. Following immobilization, the animals are immediately exposed to forced swim paradigm for 20 ​min (in a tall cylindrical tank of water). Then, the animals are left to rest for 15 ​min. Following the rest, the animals are exposed to anesthesia (with diethyl ether until loss of consciousness). The animals are then placed in their home cages and remained undisturbed for seven days to allow development of post-traumatic stress disorder symptoms (Liberzon and Young, 1997; Perrine et al., 2016).

***Early-Life Single Prolonged Stress (SPS):*** Rats are ordered as consolidated litters and allowed to acclimatize for one week. At PND21, the rats are separated from the dam and allocated into new cages. The rats are then subjected to single prolonged stress (SPS: 2 ​h restraint stress, 20 ​min forced swim stress, 15 ​min break, then 2–3 ​min anesthesia), at PND25 as explained above (Liu et al., 2017). Behavioral tests including anxiety-like behavior, learning-memory function and depression-like behavior can be conducted at PND32, PND60 and PND90 or later. Moreover, early life prolonged stress also caused co-occurrence of anxiety and depression-like behavior at early life while depression-like behavior was observed only at adulthood (Liu et al., 2017). Behavior deficits reportedly persist much later but at least until PND 90 (Liu et al., 2017).

***Deprivation Paradigms-*** Deprivation of food or water is a survival threatening stress. Food deprivation in rats produces alterations in numerous hormonal and behavioral responses compatible with those seen during stress response (Dallman et al., 1999). Similarly, water deprivation induces hormonal changes that are connected to stress induced HPA axis activation (Kiss, Jezova and Aguilera, 1994). *Sleep Deprivation*: Sleep is essential for maintenance of homeostasis and play essential role in many brain functions, therefore, sleep deprivation alters brain functions and contribute to allostatic load throughout the body (B. S. McEwen, 2006). Many animal models are used to study the psychological and the pathological consequences of acute or chronic sleep deprivation. Additionally, since there are different stages of sleep, there are different models of sleep deprivation that target a particular stage of sleep such as rapid eye movement (REM) sleep deprivation. Some models include forced activity to maintain wakefulness state, others disturb sleep by gentle handling or enriched environments. Platform method was developed to target REM sleep where the animals are placed on a narrow platforms in a water tank. Animals can sleep on the platforms, but once they enter REM sleep, the loss of muscle tone causes them to fall in the water, hence, the animals are deprived of REM sleep (K. Alkadhi, 2013). When choosing sleep deprivation method, it is important to choose a method which eliminates confounding factors such as extra physical activity or isolation stress. Pinnacle automated sleep deprivation system is a gentle method for disturbing sleep in rodents by gentle movement of a rotating bar at the base of a cylindrical cage. This system mimics gentle handling method of sleep deprivation with the advantage of restricting personnel involvement (Atrooz, Liu, Kochi and Salim, 2019). Acute sleep deprivation for 24 ​h was associated with elevation of oxidative stress and proinflammatory markers in brain tissues and blood (Solanki, Atrooz, Asghar and Salim, 2016). In animal models of chronic sleep deprivation, the animals are deprived of sleep for few hours daily over an extended period of time mimicking the human condition of late “bedtime” or early “wakeup”. Chronic sleep deprivation in rodents has been associated with memory impairment and associated with an increase in oxidative stress and free radical production, suggesting that sleep deprivation is a chronic stressor and that the resulting allostatic load can lead to different diseases and cognitive problems (B. S. McEwen, 2006). *Early-Life Sleep Deprivation*: Sleep during developmental stages of early life is suggested to play an essential role in postnatal brain development. Therefore, sleep deprivation at early life has long lasting effect on behavior and brain development (Atrooz et al., 2019). However, gentle methods should be used to induce sleep deprivation in young animals to minimize confounding factors. Pinnacle automated sleep deprivation system is used to induce sleep deprivation in rats (Pinnacle Technology, Lawrence, KS, USA). This system effectively produces sleep deprivation in rats as validated by polysomnography studies (Hines, Schmitt, Hines, Moss and Haydon, 2013; Wooden et al., 2014). The apparatus is a Plexiglas cylindrical cage with a rotating bar at the base. The bar is controlled by a software which enables controlled bar rotation at the desired speed and direction. Random bar rotation at moderate rotation speed of 10–40 rotations are recommended so that the rotating bars gently touch rat feet and disturb their sleep, while preventing adaptation to the bar rotation. The cages are layered with corn cob bedding and equipped with water bottles. At PND19, rats are subjected to sleep deprivation 6 ​h per day starting at 8:00 a.m. central time (ZT1) for 7 days. Two rats are placed in each sleep deprivation apparatus to minimize social isolation stress. After 7 days, the sleep deprivation duration is increased to 8 ​h per day for 7 additional days. This increment is necessary as the percentage of wakefulness during light phase increases with development in rats (Alfoldi, Tobler and Borbely, 1990). The control rats are placed in similar cages (two rats per cage) and left undisturbed in the same room. At the end of the sleep deprivation protocol; at PND33, behavior and cognitive tests can be conducted (Atrooz et al., 2019). Sleep deprivation during early life was associated with early induction of anxiety-like behavior and late onset of depression-like behavior, which might indicate that anxiety-like behavior precedes depression-like behavior in this model (Atrooz et al., 2019). *Social Isolation:* Social isolation involves solitary housing. Social isolation and lack of social support are the most relevant stressors that are deleterious for health in humans and also in animals. Therefore, social isolation paradigm is considered as an appropriate method for stress induction in rodents. Post-weaning social isolation includes the rearing of rats in isolated cages starting from the day of weaning ends until the day of behavioral testing, typically, until late-adolescence phase or adulthood period. Usually, in-isolation-reared rats are placed and housed within the same holding room as group-reared control rats, such that socially isolated rats are able to receive olfactory, auditory and visual cues from other rats, although they are devoid of social contact (Lapiz et al., 2001). Post-weaning social isolation in rats produces long-lasting alterations in behavior related to altered fear and anxiety, which is closely associated with changes in neuroendocrine function and the monoaminergic systems activity (Lukkes, Watt, Lowry and Forster, 2009). In adult rodents, social isolation shows some variation from post-weaning social isolation because they have previous social experience. Social isolation in adult rats can be initiated at 7–8 weeks and can last for 2–12 weeks. Social isolation studies in adult rats reported behavioral deficits such as anxiety-like and depression-like phenotypes (Ahmed, Stinus, Le Moal and Cador, 1995; Barrot et al., 2005; Wallace et al., 2009). *Early Maternal Separation Stress*: Early maternal separation can induce long-lasting effects on emotionality and stress response (Levine, 1957; Vetulani, 2013). Therefore, maternal separation in rodents, mainly rats, has been used as a model to study stress-related psychiatric disorders (Nishi, Horii-Hayashi and Sasagawa, 2014). In this paradigm, neonates are separated from dams few days after birth for a specific period before they are placed back alongside the dam. The separation is repeated daily up to 14 days; hence, this paradigm is considered as a prolonged stress model (Bhatia et al., 2011). Different groups have used different duration of maternal separation ranging from 1 to 24 ​h/day to 1–21 days (Barreau, Cartier, Ferrier, Fioramonti and Bueno, 2004; Biagini, Pich, Carani, Marrama and Agnati, 1998; Tjong et al., 2010). Maternal separation model has been used extensively in the literature to study the effect of early life stress on vulnerability to addiction and psychostimulant responsiveness at adult stage (Kosten and Kehoe, 2005; Kosten, Sanchez, Zhang and Kehoe, 2004). Rodent models of early life adversity have also been used to induce anxiety across development (Liu et al., 2017; Murthy and Gould, 2018). Maternal separation was one of the most used models. Earlier studies of maternal separation emphasized the importance of environmental factors at early life in regulating the development and the responsiveness of the HPA axis to stress. Rats and mice pups that are repeatedly separated from the dam, exhibit anxiety-like behaviors at adulthood and increased CRF mRNA level in the hypothalamus. (Plotsky and Meaney, 1993). Maternal separation in rodents, mainly in rats, has been proposed as an animal model of early life stress and for subsequent development of depression-related behaviors in adulthood (O'Mahony et al., 2009; D. A. Slattery and Cryan, 2017). Early-life short handling period (15 ​min) is reported to have beneficial effects in rodents (Levine, 1957; Pryce et al., 2005; Schmidt, Wang and Meijer, 2011). In contrast, early-life maternal separation paradigms that involve extended and/or repeated separation duration (1–24 ​h/day for 1–21 days) have been linked to development of depression-like behaviors such as anhedonia, passive coping, and also reported to have significant alterations in the neuroendocrine system (Ladd, Huot, Thrivikraman and Nemeroff, 1998; Matthews and Robbins, 2003; Matthews, Wilkinson and Robbins, 1996; Bruce S McEwen, 2003; Pryce et al., 2005).

***Environmental Stressors***: Changes in the environmental conditions such as acute temperature fluctuation, noise exposure, or exposure to predator odor, produce profound effects on stress response system in rodents. *Temperature fluctuation induced stress*: Acute change in ambient surrounding temperature leads to stressful conditions through activation of temperature regulatory center in the hypothalamus and subsequently HPA axis and stress response activation (Sapolsky, Krey and McEwen, 1986). Temperature fluctuation induced stress include exposure to low or high temperature as described. *Cold Exposure*: A sharp decrease in surrounding temperature using water or freezer has been used to induce cold stress in animals. Immersion in cold water involves placing the animals individually in a tank filled with cold water (15–20 ​°C) for 15 ​min (Retana-Marquez et al., 2003). Cold environment isolation involves placing the animals individually in a freezer with a temperature maintained at 4 ​°C for 15 ​min (Kvetnansky, Gewirtz, Weise and Kopin, 1971). Exposure to cold environment has been done in the literature by one time exposure for application of acute stress or by repeated cold exposures for 7–10 days to induce chronic stress. *Heat Exposure*: Heat stress is done by incubating the animals (rats or mice) for several hours at 38 ​°C in a biological incubator with oxygen supply. Chronic heat exposure (1 ​h/day for 21 days) caused alterations in electrical activity in rat cortex as revealed by electro-encephalogram (EEG) recordings (Sinha, 2004).

*Noise Exposure*: Noise is a stressful stimulus. Experimental studies demonstrated that noise that exceeds 90 ​dB is considered a stressor (Bhatia et al., 2011; Manikandan and Devi, 2005). Stressful noise was found to affect sodium-dependent high-affinity choline uptake in the brain. Changes in cholinergic activity in the central nervous system could be a response to the stressful effect of noise (Lai, 1987). Moreover, noise stress generates excess reactive oxygen species in the brain leading to allostatic load of oxidative stress (Manikandan and Devi, 2005). Noise stress paradigm can be produced by loudspeakers installed 30-cm above the cages. The noise level in this model was set at 100 ​dB or above throughout the cage and monitored using a sound level meter. In this paradigm, animals are exposed to noise stress in their home cages for 4 ​h/day for 15 days (Bhatia et al., 2011; Manikandan and Devi, 2005). *Predator Odor Exposure*: Exposure of rodents to their natural predator odor may induce stress-like states. This paradigm involves placing the rodents on their natural predator odor (e.g. used cat litter) for 10 ​min in a closed environment (Blanchard, Blanchard, Weiss and Meyer, 1990). Control animals are exposed to fresh, unused litter for the same duration of time. Exposure to predator odor caused rapid activation of sympathetic system and stimulation of the HPA axis collectively leading to the development of anxiety-like behavior. In fact, predator encounter-based models are widely used as models of anxiety. Exposure to predator or predator odor triggers defensive responses which resemble emotional states similar to that of fearful behaviors and anxiousness. The behavioral response of the animals exposed to predator or predator odor mimic several symptoms of PTSD, such as hyperarousal avoidance of trauma-related cues, and chronic generalized anxiety (F. Berton, Vogel and Belzung, 1998; Hebb et al., 2003). In rodent models, inescapable predator stress (exposure to a cat or cat odor) induces long lasting anxiety-like behavior as indicated by several tests of anxiety including elevated plus maze, light dark box and startle-potentiated response (R Adamec, 1997; Robert E Adamec, Blundell and Burton, 2003; Robert E Adamec, Blundell and Collins, 2001; Robert E Adamec and Shallow, 1993). Interestingly, pretreatment with the anxiolytic benzodiazepine, chlordiazepoxide, reduced anxiety-like behavior in rats following predator odor exposure (Zangrossi Jr and File, 1992). In addition, predator exposure induces changes in brain structure and function resemble that have been associated with the genesis of PTSD symptoms in humans. For example, predator exposure in rodents induced modifications in glucocorticoid levels and the CRF gene expression and release in the amygdala (R. E. Adamec, 1998; Robert E Adamec et al., 2003; Robert E Adamec, Blundell and Burton, 2005). Furthermore, predator stress induced long lasting potentiation of afferent and efferent transmission of amygdala to a degree that is highly predictive of changes in PTSD-like phenotype (Robert E Adamec et al., 2005). Moreover, the induced PTSD-like behavior was associated with extensive dendritic retraction in the hippocampus but dendritic proliferation in the basolateral amygdala (Robert Adamec, Hebert, Blundell and Mervis, 2012; Cohen, Kozlovsky, Matar, Zohar and Kaplan, 2014). Notably, bilateral stimulation of the amygdala significantly decreased the avoidance behavior in rodents when compared to animals that received no stimulation following predator odor exposure (Dengler, Hawksworth, Berardo, McDougall and Papanastassiou, 2018). Interestingly, not all animals exposed to predator paradigm exhibit PTSD-like behavior, in fact, active and passive coping rats were both observed in groups of rats exposed to predator odor. Epigenetic modifications for specific genes might partly mediate coping style (Bhattacharya, Fontaine, MacCallum, Drover and Blundell, 2019). For instance, DNA methylation of Disks Large-Associated Protein (Dlgap2) gene that encodes a postsynaptic density protein is increased in hippocampus region of animals that display an PTSD-like phenotype but not in non-PTSD-like rats following the predator stress paradigm (Chertkow-Deutsher, Cohen, Klein and Ben-Shachar, 2010). Additionally, phenotypic variability in coping with predator-induced stress was associated with the degree of methylation of the AVP in the amygdala. AVP is predominantly involved in stress adaptation and anxiety. Interestingly, rats exhibited active coping had less methylation of the AVP promoter in the medial amygdala compared to passive coping rats (Bowen et al., 2014; Choleris et al., 2009). *Electric foot shock induced stress*: Delivery of mild intensity electric foot shock has also been used as a stressor. Rodents exhibit rapid stress response even to mild shock as indicated by studies which have suggested that mild shock leads to development of behavioral and neurochemical changes which mimic depression, anxiety, and PTSD in humans (Bali and Jaggi, 2015). In rodents, electric shock is produced by placing the animals individually in a chamber fitted with an electrified grid floor. For example, rats receive unavoidable 3 ​mA intensity electric foot shocks for a duration of 200 ​ms with a frequency of one shock per second over a period of 5 ​min. Electric foot shock can be applied to induce acute or prolonged stress by following different paradigms. For acute stress response, the animals are exposed to one session of foot shock and sacrificed 15 ​min following the end of the session. For prolonged stress, animals are exposed to repeated sessions of foot shock for 7–10 days, animals can be sacrificed 1 ​h after the last session (Bhatia et al., 2011). By varying the electric shock parameters, different stress models have been created such as fear-potentiated startle and fear conditioning that reflect models of anxiety and PTSD, respectively. *Fear conditioning*: This is an example of the relationship between occurrence and experience of aversive events and the environmental stimuli that predict such events. In rodent fear conditioning paradigm, researchers typically use a tone or a light as a conditioned stimulus (CS) and a mild electric shock (e.g. a foot shock) as unconditioned stimulus (US). Repeated presentation of CS–US pairings produce robust fear learning, as a result, subsequent presentations of the CS, without pairing with US, induces fear responses (Kamin and Jones, 1968). The typical fear response indices in rats include; freezing, potentiated startle, analgesia or alterations in autonomic nervous system activities (Brown, Kalish and Farber, 1951; Davis, 1997; Fanselow, 1986; Kamin and Jones, 1968; Stiedl and Spiess, 1997). Because fear conditioning occurs very rapidly, this model has been used widely for studying the molecular substrates involved in regulation of learning and memory processes (Jeansok J Kim and Jung, 2006).

***Genetic Models:*** The aim of the genetic models is to increase stress responsiveness using advanced approaches that include genetic manipulations or selective breeding (Patchev and Patchev, 2006). Genetic manipulation is the most advanced approach to stress liability modeling, it involves modifications of the expression of targeted genes encoding specific components of stress-response cascade (Patchev and Patchev, 2006). The noradrenergic system is involved in the regulation of a variety of physiological and psychological processes, such as those involved in the modulation of the wide spectrum of moods (Ressler and Nemeroff, 1999). It has been reported that the genetic knock-out of the α_2A_-adrenergic receptors (α_2A_-ARs) predispose mice to less activity in forced swim test and more anxiety-like behavior than wild-type C57 Bl/6 mice (Schramm, McDonald and Limbird, 2001). In fact, large number of studies on anxiety have been carried out in animals with different genetic background. For example, Inbred Wistar–Kyoto (WKY) rats have been proposed as a model of increased anxiety vulnerability. WKY rats display hypervigilant state which might contribute to its anxiety vulnerability (McAuley et al., 2009). Compared to the outbred Wistar rat strain, Fawn-hooded rats exhibit more freezing behavior in response to stress and an increased preference for alcohol. Fawn-hooded rats also show increased levels of CRF mRNA in the central nucleus of the amygdala and reduced tonic HPA axis activity which may play a role in the hyperarousal characteristics of Fawn-hooded rats (Altemus, Smith, Diep, Aulakh and Murphy, 1994). Few mouse lines have been described as models of anxiety such as; the inbred BALB/c mouse, 129 mouse, and BTBR T ​+ ​tf/J mouse strains (Belzung and Griebel, 2001; Camp et al., 2009; Pobbe et al., 2011). In addition to the wide variety of genetically modified lines that have been developed by either spontaneous mutations, or by targeted candidate genes, such as CRF overexpressing mouse (Finn, Rutledge-Gorman and Crabbe, 2003; Heinrichs et al., 1997). Corticotropin-releasing hormone (CRH) is considered as a primary mediator of the neuroendocrine and behavioral responses to stress. Several studies utilizing experimentally engineered or stress-induced dysregulation of gene expression within the CRH system is reported to be associated with abnormal responses to specific environmental perturbations, which are believed to emulate clinical findings of CRH system dysregulation in several psychiatric disorders (Bakshi and Kalin, 2000). Tissue-specific mutations of glucocorticoid receptor (*Gr*) gene using the Cre/*loxP* -recombination system leads to loss of glucocorticoid receptors (GR) function. Loss of GR function in mice brain altered HPA–axis regulation, indicated by elevation of glucocorticoid (GC), and the mice exhibited symptoms similar to those observed in Cushing syndrome (Tronche et al., 1999). In another study, scientists succeeded in generating a line of mice with time-dependent knockout of GR in forebrain (FBGRKO). This type of genetically modified mice developed physiological and behavioral abnormalities that seem to closely mirror major depressive disorder in humans, also exhibit hyperactivity of the HPA axis, impaired negative feedback regulation of the HPA axis and, and demonstrated an increased depression-like behavior phenotype (Boyle et al., 2005).

Breeding strategies aim at the consolidation of behavioral traits that are associated with increased vulnerability to stress. For instance, several rat strains are characterized by enhanced anxiety and irregular behavioral responses to stress associated with increased HPA axis activity for example Fawn-Hooded, Maudsley reactive, and Roman high avoidance strains (Blizard and Adams, 2002; Kantor, Anheuer and Bagdy, 2000). Other rat strains such as Syracuse low avoidance strain show hypertrophy of the adrenal glands but reduced synthesis and release of corticosterone, the paradoxical HPA axis activity is associated with higher state and trait anxiety (Brush, 2003). The Wistar–Kyoto (WKY) rat strain considered as an animal model of endogenous depression reportedly shows hyper reactivity to stress and dysregulation of the HPA and hypothalamic–pituitary–thyroid (HPT) axes (Solberg, Olson, Turek and Redei, 2001). Large number of studies on anxiety have been carried out in animals with different genetic background. For example, Inbred Wistar–Kyoto (WKY) rats have been proposed as a model of increased anxiety vulnerability. WKY rats display hypervigilant state which might contribute to its anxiety vulnerability (McAuley et al., 2009). Compared to the outbred Wistar rat strain, Fawn-hooded rats exhibit more freezing behavior in response to stress and an increased preference for alcohol. Fawn-hooded rats also show increased levels of CRF mRNA in the central nucleus of the amygdala and reduced tonic HPA axis activity which may play a role in the hyperarousal characteristics of Fawn-hooded rats (Altemus et al., 1994). Few mouse lines have been described as models of anxiety such as; the inbred BALB/c mouse, 129 mouse, and BTBR T ​+ ​tf/J mouse strains (Belzung and Griebel, 2001; Camp et al., 2009; Pobbe et al., 2011). In addition to the wide variety of genetically modified lines that have been developed by either spontaneous mutations, or by targeted candidate genes, such as CRF overexpressing mouse (Finn et al., 2003; Heinrichs et al., 1997).

### 1.2 Stress and learning-memory functions

Although stress is a necessary process for survival, chronic stress or high plasma level of stress hormones negatively affects brain's structure and functions including learning and memory. Stress or stress hormone impairs cognitive function in animals model and humans (A. Aleisa et al., 2006; A. M. Aleisa, Alzoubi and Alkadhi, 2006a; K. H. Alzoubi et al., 2009; Gerges, Alzoubi, Park, Diamond and Alkadhi, 2004; Park, Campbell and Diamond, 2001; Srivareerat, Tran, Alzoubi and Alkadhi, 2009; Xiong and Stringer, 2000) by enhancing the adrenal corticosteroids output (J. J. Kim and Yoon, 1998; Ohl, Michaelis, Vollmann-Honsdorf, Kirschbaum and Fuchs, 2000). The effects of stress in animal studies are dependent upon the duration of stress applied. Memory testing of animal models of stress in the radial arm water maze shows that acute stress has no apparent effect on learning or long-term memory but reversibly impairs short-term memory*.* However, chronic psychosocial stress causes impairment of short term as well as long-term memory (K. H. Alzoubi et al., 2009). The effects of stress on learning and memory come about through functional alterations of brain regions important for cognitive processes, particularly the hippocampus (Diamond, Campbell, Park, Halonen and Zoladz, 2007; Joels and Baram, 2009; Nathan, Griffith, McReynolds, Hahn and Roozendaal, 2004; Ramos and Arnsten, 2007; C. Sandi and Pinelo-Nava, 2007; Segal, Richter-Levin and Maggio, 2010; A. H. van Stegeren, 2009). The hippocampal formation, an important learning and memory brain structure, is uniquely vulnerable to stress hormones due to its high expression of glucocorticoid receptors (B. S. McEwen, 1999, 2001). Reports from various laboratories show that stress impairs hippocampus-dependent spatial memory and suppresses synaptic plasticity in hippocampal area CA1 of the rat (A. Aleisa et al., 2006; A. M. Aleisa et al., 2011; K. A. Alkadhi, 2018; K. A. Alkadhi and Tran, 2014; Diamond, Fleshner, Ingersoll and Rose, 1996; Diamond, Park, Heman and Rose, 1999; Mesches, Fleshner, Heman, Rose and Diamond, 1999; C. Sandi et al., 2005; Tran, Srivareerat and Alkadhi, 2010; R. M. Vouimba, Munoz and Diamond, 2006).

### 1.3 Synaptic plasticity during stress

A foremost example of synaptic plasticity is long-term potentiation (LTP), a widely accepted correlate of learning and memory (K. A. Alkadhi and Tran, 2014; Diamond et al., 2007; J. J. Kim, Song and Kosten, 2006; Tran, Srivareerat and Alkadhi, 2011). Alterations of synaptic functions are evident through synaptic plasticity by the strengthening (i.e., LTP) or weakening (i.e., long-term depression or LTD) of synaptic transmission. Electrophysiological findings show that chronic psychosocial stress blocks the induction of LTP and enhances LTD (A. Aleisa et al., 2006; Gerges, Alzoubi, et al., 2004). For instance, in urethane-anesthetized rats, electrophysiological studies established that chronic stress significantly suppresses LTP in area CA1 of the hippocampus and facilitates induction of LTD (A. M. Aleisa, Alzoubi and Alkadhi, 2006b; L. Xu, Anwyl and Rowan, 1997). Remarkably, predator exposure stress model impairs LTP of area CA1 (R. M. Vouimba et al., 2006) but augments LTP of the dentate gyrus (DG) (Dringenberg, Oliveira and Habib, 2008). In hippocampal area CA1, increased release rate of implanted corticosterone severely impairs LTP (Diamond, Bennett, Fleshner and Rose, 1992; Gerges, Aleisa, Schwarz and Alkadhi, 2004; Pavlides, Watanabe and McEwen, 1993) whereas it shows no effect on LTP of the DG (R.-M. Vouimba and Richter-Levin, 2013)*.* Moreover, moderate doses of corticosterone enhance LTP in the DG area but not in area CA1. Strangely, in area CA, higher doses of corticosterone convert LTP to LTD without disturbing LTP of the DG of the same rat (Sharvit, Segal, Kehat, Stork and Richter-Levin, 2015). Furthermore, corticosterone inhibits local circuit plasticity induced by theta burst stimulation only in the DG area (Sharvit et al., 2015). Interestingly, acute stress significantly enhances LTP in the amygdala (Maroun, 2006; R. M. Vouimba et al., 2006; R. M. Vouimba, Yaniv, Diamond and Richter-Levin, 2004) a brain region that is critically involved in the processing of emotional memory (LeDoux, 2000; J. L. McGaugh, 2004). Stress or a fear-provoking experience activates the amygdala (Ilin and Richter-Levin, 2009; Monfils, Cowansage and LeDoux, 2007; Pare, 2003), which in turn exerts an inhibitory effect on hippocampal plasticity (Akirav and Richter-Levin, 1999, 2006). In contrast, acute stress experiments have revealed the vulnerability of area CA1 to impairment of synaptic plasticity, (A. M. Aleisa et al., 2006a; K. A. Alkadhi, 2018; K. A. Alkadhi and Tran, 2014; Alzoubi, Srivareerat, Tran and Alkadhi, 2013; Diamond et al., 2007; Gerges, Aleisa, et al., 2004; Joels and Krugers, 2007; J. J. Kim et al., 2006; Tran et al., 2011).

Stress affects synaptic plasticity differently in different regions of the hippocampal formation differently. Stress hormones and stress impair LTP (A. M. Aleisa et al., 2006b; Pavlides, Ogawa, Kimura and McEwen, 1996; R. M. Vouimba et al., 2006; L. Xu, Holscher, Anwyl and Rowan, 1998) and enhances LTD (K. A. Alkadhi and Tran, 2014) in hippocampal area CA1, whereas the LTP of DG in the same animals seems to be more resistant to stress and stress hormones (A. M. Aleisa et al., 2006a; Bramham, Southard, Ahlers and Sarvey, 1998; Gerges, Stringer and Alkadhi, 2001). Electrophysiological recording from anesthetized rat brain shows that while chronic psychosocial stress does not affect LTP of the DG, it prevents the expression of LTP in area CA1of the same rat. To explore the possible mechanism for this differential effect, we revealed that although chronic stress significantly increases the phosphatase calcineurin level area CA1, in the DG, stress produces a significant decrease in calcineurin levels of the same rat. These findings show that in the chronically stressed rats, only the DG is endowed with a defense mechanism that curtails dephosphorylation to maintain normal level of the synaptic plasticity-essential phosphorylated-CaMKII molecule. This we consider to be responsible for the normal LTP of the DG of chronically stressed rats (Gerges, Aleisa, Schwarz and Alkadhi, 2003). It is conceivable that the resistance of the DG area to the effects of stress is probably due, in part, to the capacity of the DG area to replaced dysfunctional neuron through neurogenesis. The existence of this defense mechanism in the DG area is probably related to maintaining the quintessential ability of this area for neurogenesis.

### 1.4 Neurogenesis and stress

Studies in various animal stress models indicate that stress and stress hormones can markedly suppress neurogenesis. Neurogenesis occurs throughout life in certain areas of the brain including the subgranular zone of the DG area, subventricular zone and olfactory bulb (Mirescu and Gould, 2006; Tawarayama, 2018). Chronic restraint stress inhibits adult hippocampal neurogenesis in mice through inducing autophagic cell death (ACD) of hippocampal neural stem cells (Jung et al., 2020). The use of transgenic animal models plays a central role in enabling more extensive study of the behavioral function of adult neurogenesis. Early work showed that stress and stress hormones suppressed cell proliferation in the adult dentate gyrus (Gould, Cameron, Daniels, Woolley and McEwen, 1992; E. Gould et al., 1997, Gould et al., 1997). The newborn neurons express both glucocorticoid and mineralocorticoid receptors (Cameron, Woolley and Gould, 1993). Experiments reported by Du Preez and colleagues (2021) show animals undergoing unpredictable chronic mild stress (UCMS) followed by social isolation reveal significantly reduced neurogenesis in the DG area as well as reduced levels of peripheral vascular endothelial growth factor (VEGF), which is a trophic factor that stimulates neurogenesis (Du Preez et al., 2021). The use of bromodeoxyuridine and doublecortin immunostaining method to detect neurogenesis revealed significantly fewer labeled cells in the DG area of animals exposed to predator stress, indicating interference in neurogenesis (Wu et al., 2019).

### 1.5 Brain morphology and stress

Abundant studies indicate that in addition to impairing performance on hippocampus, amygdala and prefrontal cortex dependent memory tasks, stress induces morphological alterations within each brain region (C. D. Conrad, 2006; Holmes and Wellman, 2009; J. J. Kim & D. M. Diamond, 2002; Liston, McEwen and Casey, 2009; Lupien, Maheu, Tu, Fiocco and Schramek, 2007; B. S. McEwen, 2007; Zoladz, Fleshner and Diamond, 2012)*.* Stress responses are mediated by two essential components of the brain's neural circuitry namely the hippocampus and the amygdala. Two different models of chronic stress have been used to study the effects of stress on hippocampal and amygdaloid neuronal morphology in rats (Vyas, Mitra, Shankaranarayana Rao and Chattarji, 2002). Chronic immobilization stress caused dendritic atrophy and loss of branching in CA3 pyramidal neurons of the hippocampus*.* In contrast, the same stress model caused enhanced dendritic branching in the pyramidal and stellate neurons of the basolateral amygdala (Daskalakis, De Kloet, Yehuda, Malaspina and Kranz, 2015; Vyas et al., 2002). However, in the second model of stress, chronic unpredictable stress, did not affect CA3 pyramidal neurons but induced atrophy only in basolateral amygdala bipolar neurons (Vyas et al., 2002). Thus, the outcome of stress in the brain depends both on type of stress model and the brain region. Structural changes are the result of alterations in dendritic morphology and spine remodeling, whereas functional alterations are manifest through the strengthening (i.e., LTP) or weakening (i.e., LTD) of the synaptic function*.* Stress does not only impact neurons, it also affects other cells in the brain. For example, stress and corticosteroid are known to cause major structural remodeling of microglia and enhances the release of pro-inflammatory mediators (van Olst, Bielefeld, Fitzsimons, de Vries and Schouten, 2018). Corticosteroids have been shown to affect the structure of hippocampal synapses. Based on optical imaging of dendritic spines, vividly rapid dendritic spine modulation is seen after applying corticosterone, which is probably operating through activation of mineralocorticoid receptors (Gulyaeva, 2019; Murakami et al., 2018). This structural modification together with functional changes have been proposed to be a cellular basis of learning and memory (Matsuzaki, Honkura, Ellis-Davies and Kasai, 2004). The effects on brain structure often depend on whether stress is acute or chronic as well as on the specific area of the brain being examined. The majority of chronic stress research has focused on describing changes in morphology of primarily area CA3 (McEwen (B. S. McEwen, 2007; McLaughlin, Gomez, Baran and Conrad, 2007) or on the process of neurogenesis in the dentate gyrus (Fuchs, Flugge and Czeh, 2006; P. Lucassen et al., 2010). It has been reported that chronic restraint stress decreases dendritic spine density in area CA3 of the hippocampus, while increasing spine density in the basolateral amygdala (Daskalakis et al., 2015). These changes seem to implicate brain derived neurotrophic factor (BDNF), which is an important nurturer of the structural and functional integrity of neurons. For example, it has been shown that chronic restraint stress increases BDNF mRNA and protein levels in the basal lateral amygdala but decreases these levels in the CA3 area of the hippocampus. These stress-induced changes in the BDNF are accompanied by intensifications of dendritic spine density in the amygdala and decreases it in area CA3 (Daskalakis et al., 2015).

### 1.6 Molecular targets of stress

Although various reports have shown that memory and stress modify plasticity-related protein molecules expression (Bisaz and Sandi, 2010; Izquierdo, Wellman and Holmes, 2006), fewer studies have compared molecular plasticity in animals having intact memory with those rendered amnestic by stress. Stress, especially chronic stress, can damage the structure and function of the brain. However, the brain is quite capable of adaptive plasticity and resilience (B. S. McEwen and Akil, 2020). Based on studies in animal models, numerous systemic and neural factors, which can gain access to the brain, contribute to this adaptive plasticity*.* These factors include BDNF (de Assis & Gasanov, 2019), insulin; IGF-1 from liver (Trejo, Carro and Torres-Aleman, 2001); a muscle hormone, cathepsin B (Moon and van Praag, 2014) and the bone hormone osteocalcin (Khrimian et al., 2017), which are engaged in various neuroprotective processes including neurogenesis, dendritic growth and synaptogenesis (Lu, Nagappan, Guan, Nathan and Wren, 2013; Sasi, Vignoli, Canossa and Blum, 2017). It is also well known that stress impairs expression of BDNF in the hippocampus (A. Aleisa et al., 2006; Alzoubi et al., 2013; Duman and Monteggia, 2006; Radecki, Brown, Martinez and Teyler, 2005; Zoladz et al., 2012). As indicated earlier, chronic restraint stress increases BDNF mRNA and protein levels in the basal lateral amygdala but in the same model, stress decreases these levels in area CA3 of the hippocampus. Consequently, these changes in the BDNF lead to increases in dendritic spine density in the amygdala and reductions in spine density in area CA3 (Daskalakis et al., 2015), which underscores the fundamental role of BDNF in maintaining the integrity of the synaptic structure.

Stress has a profound impact on the molecular mechanisms responsible for synaptic plasticity (K. A. Alkadhi and Tran, 2015; Alzoubi et al., 2013; Bisaz, Conboy and Sandi, 2009; Gerges et al., 2003; Gerges, Aleisa, et al., 2004; Howland and Wang, 2008; Jeansok J Kim and Jung, 2006; Pittenger and Duman, 2008; Zoladz et al., 2012). Protein phosphorylation and dephosphorylation are critical cellular mechanisms that regulate various signaling molecules via kinases and phosphatases*.* In particular the Ca^2+^-dependent kinases and phosphatases tightly control the molecular mechanisms of learning and memory (Khan, Kulasiri and Samarasinghe, 2021)*.* For example, in the rat hippocampus, stress reduces the levels of phosphorylated calcium calmodulin kinase II (p-CaMKII), a signaling molecule critical to LTP induction and memory formation (K. A. Alkadhi and Tran, 2015). However, stress increases the expression of the phosphatase calcineurin (K. A. Alkadhi and Tran, 2015; Gerges et al., 2003; Gerges, Aleisa, et al., 2004; Zoladz et al., 2012). Additionally, Stress negatively impacts other signaling molecules involved in the CaMKII molecular cascade including calmodulin and membrane associated PKC in rat model of chronic psychosocial stress (Gerges, Aleisa, et al., 2004). Several other molecules are also impacted by stress; for example, Sandi and colleagues (2005) have reported that the predator stress-induced impairment of hippocampus-dependent memory was associated with a rapid reduction of levels of hippocampal neural cell adhesion molecules (NCAMs). These protein molecules are critically involved in forebrain development and synaptic plasticity (C. Sandi et al., 2005).

### 1.7 Mechanism of stress-induced impairment of the nervous system

Mental stress is known to impair neuronal excitability, neurochemistry, and structural as a well as the functional plasticity of the hippocampus (B. S. McEwen, 1999)*.* Numerous studies in rodent models of stress and in humans have revealed the damaging effects of psychosocial stress on synaptic plasticity and hippocampus-dependent learning and memory. (Diamond et al., 1999; Gerges, Alzoubi, et al., 2004; Srivareerat et al., 2009; Touyarot, Venero and Sandi, 2004); (Lupien et al., 2007). For example, extreme levels of glucocorticoids can cause deleterious modifications in hippocampal excitability, LTP and learning and memory (Alfarez, Wiegert and Krugers, 2006; P. J. Lucassen et al., 2006)*.* Stress activates the hypothalamic–pituitary–adrenal (HPA) axis with the subsequent release of corticosteroids into the blood stream (B. S. McEwen, 1999; Pavlides et al., 1996)*.* The normal level of circulating corticosteroids is relatively low and at that level it is sufficient to promote memory and increase LTP magnitude by activating the mineralocorticoid receptors*.* In contrast, under stressful conditions the circulating corticosteroids level is high enough to activate the glucocorticoid receptors, which cause impaired memory and suppression of LTP (B. S. McEwen, 1999; B. S. McEwen and Akil, 2020; Pavlides et al., 1996).

Various hypotheses developed from animal models of stress and human clinical studies. One such hypothesis, supported by animal studies in aged rats, is the “glucocorticoid cascade” hypothesis, which suggests that there is an association between frequent exposures to high glucocorticoid levels and hippocampal atrophy (Sapolsky, 1999)*.* Later hypotheses propose a more complex interactions between corticosterone and stress than the initial hypothesis (Landfield, Waymire and Lynch, 1978)*.* To explain the mechanism by which glucocorticoid reduces hippocampal volume, a “neurotoxicity” hypothesis was advanced (Gilbertson et al., 2002). This hypothesis is based on the premise that prolonged exposure to glucocorticoids impairs the capacity of neurons to overcome injurious effects, thus becoming more prone to be damaged (Gilbertson et al., 2002).

## 2 Summary and conclusion

Stressful life events often lead to the onset of severe psychopathology and occurrence of endocrine dysfunction in humans. Moreover, vulnerability to a compromised psyhcopathology and endocrine impariment is postulated to be increased by exposure to stress or experiences of severe adversity during childhood due to child neglect, child abuse, or facing or witnessing traumatic events. Various rodent models of stress have provided important insights from the context of their potential relevance to human psychopathology. Such models have proved to be quite valuable in terms of providing novel insights and examining the effects of stress on the animal behavior, endocrine function and/or immune status. Animal models involving genetic manipulations or psychosocial interventions reportedly cause adverse effects on key outcome measures that may be transient or may persistent for a longer period of time depending on the species, sex, and other experimental conditions, often causing long-lasting neurobiological effects. We conclude that studies of stress in rodents can model many features of stress that are prevelant in human populations. Importantly, animal stress studies offer a reliable window to help to elucidate the mechanisms underlying the role of stress in human psychopathology.

## CRediT author statement

Fatin Atrooz, Samina Salim and Karim Alkadhi together wrote the first draft of this manuscript and also helped with the revision of this manuscript. Samina Salim designed the over-all article and provided feedback and edited the final version of the review article.

## Declaration of competing interest

The authors declare that they have no known competing financial interests or personal relationships that could have appeared to influence the work reported in this paper.
