# Supplementary material for: Understanding stress: Insights from rodent models
Source: Curr Res Neurobiol. 2021 May 23;2:100013. doi: 10.1016/j.crneur.2021.100013 (PMC9559100; doi:10.1016/j.crneur.2021.100013)
Supplement: Supplementary file 2 [file mmc2.docx]

*
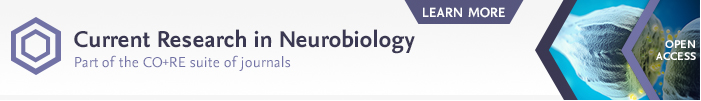
*

**Author Q&A:** Understanding Stress: Insights from Rodent Models

CRNEUR-D-21-00009R2

**Could you tell us a little bit about the basis for your study and how it helps to advance the scientific field?**

Stress is one of the most identifiable psychological phenomenon. The biological sequelae it initiates is extensive engaging multiple systems within our body, implicating the immune system, the endocrine system, the central nervous system, the reproductive system, the digestive system- practically all major systems of our body. Therefore, it is not a surprise that stressful stimuli impact our overall health and well-being. Unfortunately, in terms of therapeutic intervention, options are limited and pretty much confined to SSRIs, anti-anxiety and sleep aids. For a more precise intervention to tackle stress-related conditions, stress responses must be fully understood. Animal models can offer useful insights in this regard. This review article focused on the information animal models have offered through the work of two labs (Alkadhi and Salim), and that of the extensive network of other behavioral neuroscientists, can be used to probe and design improved therapeutics.

**What were the scientific or other challenges that you faced and how did you overcome them?**

I have combined and summarized answers to this question with the one below

**Readers might be interested in aspects that go beyond the scientific paper published. For instance, is there something about your perseverance individually or the team that you think made it possible to succeed with your research? Did you benefit from having a diversity of perspectives as part of the research either from your team or beyond?**

This question has prompted me to pause and think about myself which led to an interesting reflection on my life. My clueless teenage years, and memories of confused and unfocused college days. As I finished college, I was exhausted of the monotony of my aimless life. I enrolled in the Ph.D program in India. Graduate program was a great learning experience. It transformed a lazy and unmotivated young woman into a hard-working and ambitious person. The Ph.D. program taught me many things including the significance of **failures**, **accountability**, **patience** and **perseverance**. Following the award of my Ph.D degree, I decided to step out of my comfort zone and moved to Stockholm, Sweden in Europe to pursue post-doctoral work. The transition from a highly conservative environment to an extremely liberal Scandinavian culture was not a smooth one. As opposed to my country of birth-India, which is full of sun and full of people, Stockholm was cold, and dark and people were reserved and few. I was lonely and sad. The research program at Karolinska Institute was intense and the training was rigorous, which helped promote my growth as a scientist. As my education and training advanced, so did my ambition and pursuit for knowledge. After 3 years of post-doctoral training in Stockholm, I arrived in Houston, USA, and started working as a post-doctoral research fellow at a premier institution. This experience was not without challenges, as I did not experience the best work-place environment. I was disappointed, yet I worked very hard and focused on the objective to succeed. I learned that no one can make me feel incapable or inadequate, unless I make the decision to accept I am incapable. I learned to **value myself** and refused to be treated with anything but **respect**. I focused on my work and became all the more committed to excellence.

Soon I got a research track faculty position at the University of Houston. This position brought its own challenges. In a short time, I had to prove my worth, so I worked twice as hard as my peers. I was able to accomplish a great deal, yet it was not enough to secure a tenure-track position. I kept writing grants and publishing papers. I was beginning to doubt myself, but I ploughed through. Finally, in 2012 I was awarded a grant from the National Institutes of Health. This was the turning point of my career and I was hired as a tenure-track Assistant Professor at the University of Houston. Eventually I climbed up the ladder, became tenured and now I am an Associate Professor at the College of Pharmacy, University of Houston. This position has given me the opportunity to grow, to understand, to lead, to fail and rise again. One of my favorite things to do as part of my job is mentoring students. I feel I am able to transform their lives by giving them direction and a meaningful university experience, something I had to find myself the hard way. My position allows me to travel to new places and cultures, something I truly enjoy and cherish. Meeting new people, experiencing new cultures and seeing diversity is a beautiful experience. I am grateful to the education I received which has transformed my life and has allowed me the opportunity to explore the world. Finally, I will summarize my journey with the thought that education allows us to **practice** freedom, to **accept** change, to **embrace** criticism, to **uplift** others and become **part**of a bigger transformation.

**Are there any insights that you would like to share with other investigators or those thinking about whether to dedicate their careers to studying the brain? How do you think your work could also encourage more individuals from under-represented backgrounds to get involved in neuroscience?**

The beauty of exploring the brain is that it helps in our understanding of our own minds, our communities and the society as a whole. I know that my co-authors Drs Atrooz and Alkadhi would agree that studying the brain has been a fulfilling experience for us all. The most exciting part of studying the brain is that one could contribute and advance an approach that situates the topic of mental health within a neuro-socioecological context. In doing so, one could for example, incorporate methodologies and conceptual apparatus from various disciplines to target mental health from vantage points of basic neuroscience, ethnic and racial contexts of communities and society. The scope and the breadth offered by neuroscience research are tremendous. The wide scope allows one to connect and collaborate with the civil society, national and local non-profit organizations.

**Did you take advantage of some of our journal options (like double blind review) and how did you find the transparent review option?**

Very timely and rigorous review (single-blinded).

**Brief author bio and pictures, if you choose:**


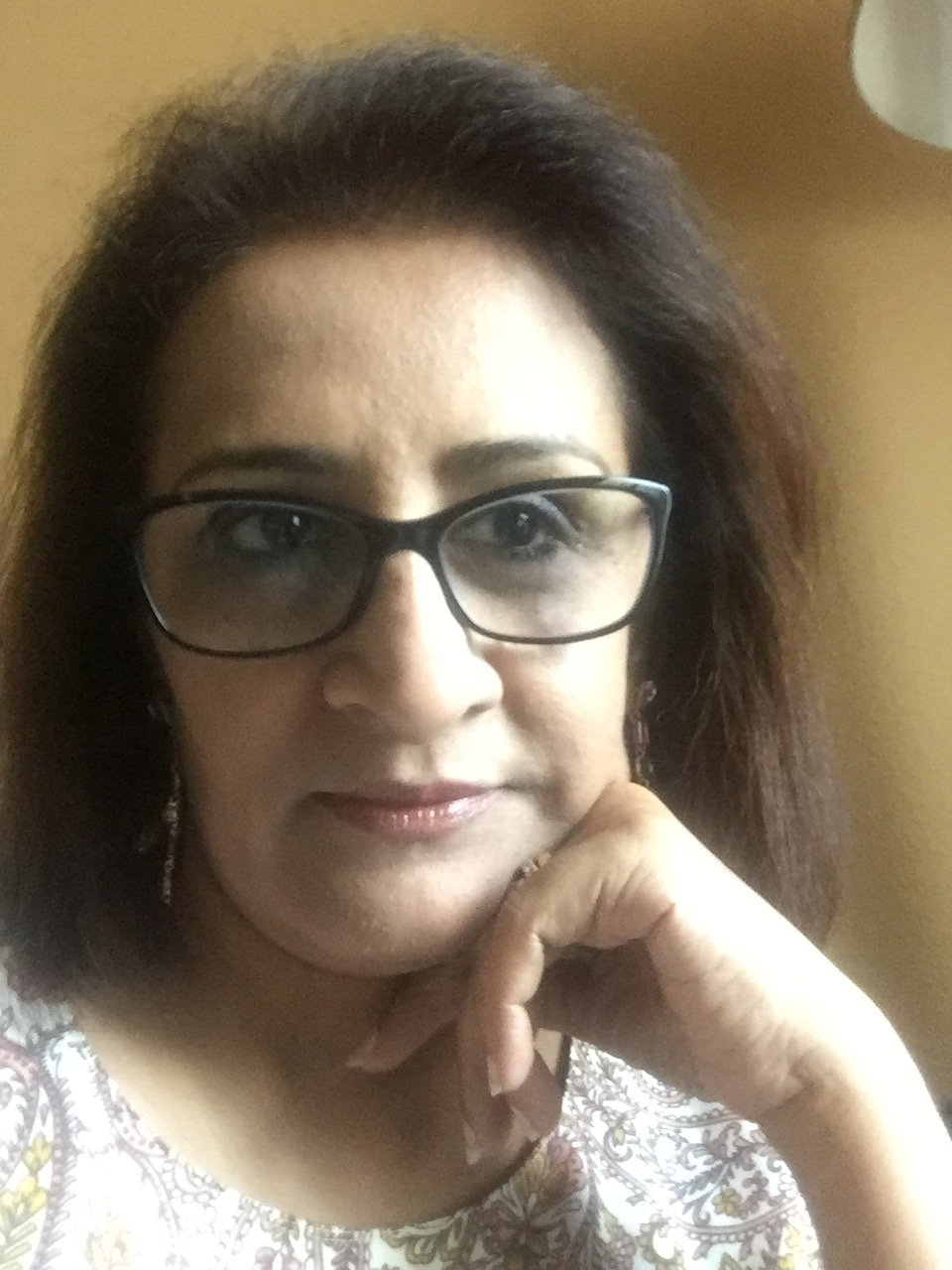


Samina Salim, PhD

My life-long goal is to study and advance the topic of health by incorporating conceptual apparatus and methods from various disciplines to target health from diverse vantage points.

*Congratulations on your scientific achievement and thank you for helping CRNEUR to continue to innovate.*

*The CRNEUR Editorial Team*
