# Supplementary material for: Understanding stress: Insights from rodent models
Source: Curr Res Neurobiol. 2021 May 23;2:100013. doi: 10.1016/j.crneur.2021.100013 (PMC9559100; doi:10.1016/j.crneur.2021.100013)
Supplement: Supplementary file 1 [file mmc1.pdf]

Peer Review Overview

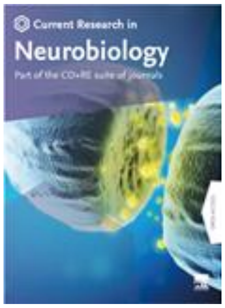

Manuscript Title: Understanding Stress: Insights from Rodent Models

Received	Feb 24, 2021
1st Decision	Apr 07, 2021
1st Revision Submitted	Apr 21, 2021
2nd Decision	Apr 28, 2021
2nd Revision Submitted	Apr 30, 2021
Accepted	May 08, 2021

1st Decision letter

Reference: CRNEUR-D-21-00009
Title: Understanding Stress: Insights from Rodent Models
Journal: Current Research in Neurobiology

Dear Dr. Salim,

Thank you for submitting your manuscript to Current Research in Neurobiology.

I have completed my evaluation of your manuscript. The reviewers recommend reconsideration of your manuscript following minor revision and modification. I invite you to resubmit your manuscript after addressing the comments below. Please resubmit your revised manuscript by May 07, 2021.

When revising your manuscript, please consider all issues mentioned in the reviewers' comments carefully: please outline every change made in response to their comments and provide suitable rebuttals for any comments not addressed. Please note that your revised submission may need to be re-reviewed.

Current Research in Neurobiology values your contribution and I look forward to receiving your revised manuscript.

Kind regards,

Anna S Mitchell, Ph.D.
Editor in Chief
Current Research in Neurobiology

Comments from Editors and Reviewers:

Two reviewers and I have reviewed your manuscript and our suggested revisions are outlined below.

The graphical abstract needs to be revised. Please remove the extended text in the graphical abstract.
The guidelines are detailed here - www.elsevier.com/graphicalabstracts

Reviewer #1:

This review article article manuscript deals with rodents model used to investigate stress phenomena.
This is indeed an important topic. The review is comprehensive and well written.

Reviewer #2:

This is an excellent review discussing several rodent models of stress paradigms and their effects on behavior and neurobiology. The following points could be considered in the revision.
The graphical abstract needs to be modified.
A revised abstract summarizing the topic of discussion would strengthen the article.
Several typos throughout the manuscript.
References are missing at many places.
"Detailed description of fear conditioning model is provided". Is the manuscript missing any Table?
The rodent models are repeatedly discussed in different sections. For example: social defeat stress and its effects on behavior have been repeatedly discussed.
Grouping the reports on the effects of different stress paradigms on behavior and neurochemical measures would make it easier for the reader to follow the content.
A careful editing of the text would shorten the length of the article and improve the quality of the manuscript.
A section on the effects of various stress models on inflammatory markers would strengthen the review.

1st Author Response Letter

Response to comments from Editors and Reviewers:

We are thankful to the reviewers for a thoughtful review of our manuscript. The suggested edits, points of clarification and other errors as pointed out by the reviewers have been addressed in the revised version of our manuscript. We greatly appreciate the time and effort invested to produce this detailed review. Our point-by-point response is provided below.

Comments from Reviewer 1

This review article-manuscript deals with rodents model used to investigate stress phenomena. This is indeed an important topic. The review is comprehensive and well written. Response: Thank you.

Comments from Reviewer 2

This is an excellent review discussing several rodent models of stress paradigms and their effects on behavior and neurobiology. The following points could be considered in the revision.

1. The graphical abstract needs to be modified.
Response: The graphical abstract has been modified to make it less textual, more illustrative and directly related to the content discussed in the review article.
2. A revised abstract summarizing the topic of discussion would strengthen the article.
Response: Done as recommended.
3. Several typos throughout the manuscript.
Response: We apologize for typographical errors. We have done a thorough editing of the manuscript and have fixed all errors and typos.
4. References are missing at many places.
Response: Added as recommended.
5. "Detailed description of fear conditioning model is provided". Is the manuscript missing any Table?
Response: This was a typo and has been corrected.
6. The rodent models are repeatedly discussed in different sections. For example: social defeat stress and its effects on behavior have been repeatedly discussed.
Response: We have removed redundancies as suggested. Detailed Response to Reviewers
7. Grouping the reports on the effects of different stress paradigms on behavior and neurochemical measures would make it easier for the reader to follow the content.
Response: This was an excellent suggestion. Done as recommended. We have extensively reorganized several sections to make the review article more cohesive with improved readability.
8. A careful editing of the text would shorten the length of the article and improve the quality of the manuscript.
Response: We have done a thorough editing of the manuscript and have trimmed it as needed.
9. A section on the effects of various stress models on inflammatory markers would strengthen the review.
Response: This is a great suggestion but we have published two comprehensive review articles recently in which this topic was extensively discussed, (Atrooz F, and Salim S. Sleep deprivation, oxidative stress and inflammation. Adv Protein Chem Struct Biol. 2020;119:309- 336; Atrooz F, Liu H, Salim S. Stress, psychiatric disorders, molecular targets, and more. Prog Mol Biol Transl Sci. 2019;167:77-105) therefore, we have not included this aspect in our discussion.

2nd Decision letter

Reference: CRNEUR-D-21-00009
Title: Understanding Stress: Insights from Rodent Models
Journal: Current Research in Neurobiology

Dear Dr. Salim,

Thank you for submitting your manuscript to Current Research in Neurobiology.

Thank you very much for suitably addressing the reviewers comments and revising your graphical abstract.

During the process of our further evaluations of your manuscript, the Editorial team have check the similarity of your review article with previous publications. I've attached the similarity report. Please can you rewrite the sentences in your manuscript that have been highlighted as showing too much overlap with previously published papers. I invite you to resubmit your manuscript after addressing

these similarities. Please resubmit your revised manuscript by May 28, 2021.

When you submit the revised manuscript, please provide a separate document, uploaded as 'Detailed Response to Reviewers,' that carefully details, point-by-point, the list of changes made in response to the reviewers' comments. You may also include a suitable rebuttal to any specific request for change that you have not made. The journal strongly encourages you to submit two versions of your revised manuscript in order to facilitate the evaluation process: one with changes in the manuscript marked (upload as file type: Manuscript (REVISED, text with changes Marked)), in addition to an unmarked, production-ready version (required) (upload as file type: Manuscript).

Current Research in Neurobiology values your contribution and I look forward to receiving your revised manuscript.

Kind regards,

Anna S Mitchell, Ph.D.
Editor in Chief
Current Research in Neurobiology

Response to comments from Editors and Reviewers:

Comment: Please can you rewrite the sentences in your manuscript that have been highlighted as showing too much overlap with previously published papers.

Response: We have revised our manuscript by removing or modifying areas of high similarity. We also have run the manuscript (excluding the reference list) through a software and achieved acceptable level of similarity. Similarity is contributed due to extensive abbreviations, references within the document and commonly used terms such as post-traumatic stress disorder, brainderived neurotrophic factor (BDNF), Hypothalamic-pituitary-adrenal (HPA) axis, depression-like behavior etc. These terms are abundantly used in the article. Another source of similarity is our previous work on the subject with identical terms which cannot be re-worded. We have however tried to address this as best as we could.

Accept Letter

Dear Dr. Salim,

Thank you for submitting your manuscript to Current Research in Neurobiology.

I am pleased to inform you that your manuscript has been accepted for publication - Congratulations.

Your accepted manuscript will now be transferred to our production department. We will create a proof which you will be asked to check, and you will also be asked to complete a number of online forms required for publication. If we need additional information from you during the production process, we will contact you directly.

We appreciate and value your contribution to Current Research in Neurobiology. We regularly invite authors of recently published manuscript to participate in the peer review process. If you were not already part of the journal's reviewer pool, you have now been added to it. We look forward to your continued participation in our journal, and we hope you will consider us again for future submissions.

Finally, if you have a chance, please consider reading our recent editorial and answering our survey. https://www.sciencedirect.com/science/article/pii/S2665945X21000012

Kind regards,

Anna S Mitchell, Ph.D.
Editor in Chief
Current Research in Neurobiology

-------- End of Review Comments --------
